# Stretching and Releasing of Iliotibial Band Complex in Patients with Iliotibial Band Syndrome: A Narrative Review

**DOI:** 10.3390/jfmk8020074

**Published:** 2023-06-04

**Authors:** Manca Opara, Žiga Kozinc

**Affiliations:** 1 Faculty of Health Sciences, University of Primorska, Polje 42, SI-6310 Izola, Slovenia; 2 Andrej Marušič Institute, University of Primorska, Muzejski trg 2, SI-6000 Koper, Slovenia

**Keywords:** iliotibial band syndrome, iliotibial band, stretching, foam rolling, knee pain, running injuries

## Abstract

Iliotibial band syndrome (ITBS) is one of the most common overuse syndromes causing knee pain; it is especially prevalent in runners and also common in cyclists, rowers, and field athletes, with occasional cases occurring in non-athletes too. ITBS symptoms can negatively affect not only knee function, but also mental and physical aspects of health-related quality of life. Although various conservative treatment options have been investigated and discussed, there is still no consensus on a standard of care for ITBS. Moreover, the literature on the etiology and risk factors of ITBS, which could help in selecting appropriate treatment methods, is conflicting and inconclusive. The role of individual treatment modalities such as stretching and releasing techniques has not been extensively studied and remains unclear. In this article, we will critically review the available evidence for the benefits of ITB stretching and “release” methods in the treatment of ITBS. In addition to the direct evidence (clinical studies examining the effects of ITB stretching and other methods that purportedly stretch or “release” the ITB), we present several additional lines of reasoning that discuss the rationale for ITB stretching/releasing in terms of the etiology of ITBS, the mechanical properties and behavior of the ITB, and the risk factors for ITBS development. We conclude that the current literature provides some evidence for the inclusion of stretching or other “release” methods in the early rehabilitation of ITBS. Long-term interventions typically include ITB stretching; however, it remains unclear to what extent stretching within a multimodal treatment actually contributes to resolving the symptoms. At the same time, there is no direct evidence to suggest that stretching and “release” methods have any negative effects.

## 1. Introduction

Iliotibial band syndrome (ITBS) is one of the most common overuse syndromes causing knee pain, and is particularly common in runners [1]. For example, in a pool of 2000 runners, 168 cases of ITBS were reported over a 2-year period, exceeded only by the prevalence of patellofemoral pain syndrome [2]. Reports of prevalence in runners are quite variable, ranging from 1.6% to 14% [1,3,4]. ITBS is also common in other sports, such as cycling [5], field sports [6] and rowing [7], with occasional cases in non-athletes [8]. While ITBS is primarily treated conservatively, the reported cure rate is modest [9], and in some cases surgery is required to resolve the problem [10]. The literature on ITBS, including its etiology [11,12,13], risk factors [11,14,15] and treatment [10,16], is conflicting and inconclusive. 

Patients with ITBS typically report lateral knee pain, localized to the area between the Gerdy tubercle and the lateral epicondyle [17]. Initially, the pain often occurs upon completion of a repetitive flexion–extension exercise. However, as the condition progresses, symptoms may occur earlier during athletic activity and even at rest [18]. In addition, running downhill or outdoors on a curved surface, lengthening the stride, and sitting for long periods of time with the knee in a flexed position often exacerbate pain [17,18,19]. Existing symptoms can negatively impact not only knee function, but also psychological and physical aspects of health-related quality of life [20].

Although various conservative treatment options have been studied and discussed, there is not yet a consensus on a standard of care for ITBS [9,16]. While the efficacy of complementary or alternative treatments such as dry needling [21], shock-wave therapy [21] and kinesiotaping [22] have recently shown some potential, the mainstay of ITBS rehabilitation includes rest or activity modification, iliotibial band (ITB) stretching, and hip abductor strengthening [16,23]. However, the role of individual exercise modalities (e.g., stretching) has not been extensively studied and remains unclear. A recent pilot study reported similar effects of ITBS stretching, conventional exercise and hip strengthening on pain and lower limb function in female runners with ITBS [24], whereas others examined the effects of a comprehensive (multimodal) program [10,25].

Recently, Friede et al. [16] provided a critical evaluation of current treatment goals in ITBS patients. While they concluded that individually tailored hip strengthening is a key component of ITBS rehabilitation, less emphasis was placed on the methods used for the stretching and releasing of the ITB. The primary aim of this article is to critically review the available evidence on the benefits of ITB stretching and release methods for the treatment of ITBS. To provide the readers with a comprehensive overview of the relevant literature, we will first present the most common methods to stretch or “release” the ITB, and discuss several additional lines of reasoning related to the rationale for inclusion of ITB stretching and “release” methods. These will include the etiology of ITBS, the mechanical properties and behavior of the ITB during stretching, as well as discussions of the risk factors for ITBS development and how these could be affected by stretching or releasing the ITB. We hope that this article will help identify critical gaps in the literature and encourage further research on the effects of stretching in the rehabilitation of ITBS. At the same time, we believe that this paper will help clinicians make better evidence-based decisions for ITBS treatment. 

## 2. Methods

To retrieve the relevant articles, we used the search term *iliotibial band AND (stretching OR release OR foam rolling OR massage)* in the PubMed, PEDro and Scopus databases. During this phase, both authors independently performed the search, and any disagreements about the inclusion of the articles were resolved by discussion. Additional papers were included if identified during the examination of the articles already retrieved. In addition, the reference lists of all systematic reviews on the topic of ITBS that were identified during the search were screened. Pertaining to the main research question, we included the articles that: (a) examined acute or chronic effects of starching, foam rolling or other “release methods”; (b) examined either ITBS patients or healthy participants; (c) compared the effects to other treatment modalities or no intervention; (d) were peer-reviewed and published in English language. Conference abstracts, editorials, opinion papers and preprints were excluded. The articles were examined via a form of narrative–qualitative synthesis. 

To provide an exhaustive review of the relevant literature, we also summarized the articles that examined in vivo or in vitro behavior of ITB during stretching or “release” methods, and provided rationale for or against the use of these modalities from the perspective of ITBS etiology and risk factors. 

## 3. Methods of Stretching and Releasing ITB–TFL Complex

**Figure 1:**
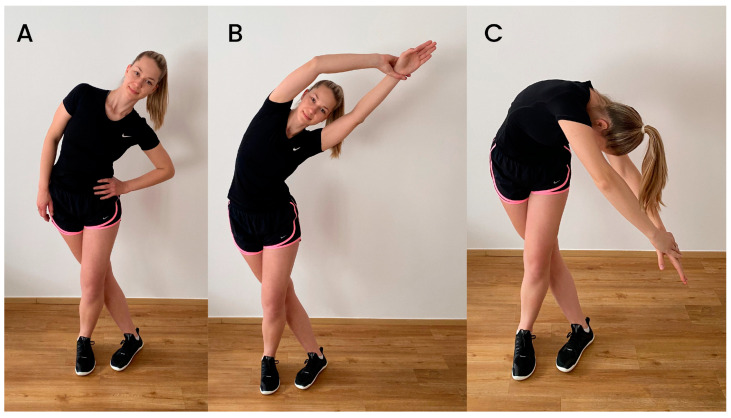
Examples of most common ITB stretches. The first stretch (**A**) involves lateral flexion of the trunk with legs crossed. In the second stretch (**B**), the arms are clasped overhead and moved sideways as well. The last example (**C**) includes a diagonal and downward movement of the trunk.

There are quite a few different stretching exercises that target the ITB–TFL complex. Combined with unclear guidelines regarding ITBS treatment, this is probably the reason why stretching exercises used in interventions studies are also diverse. Stretching exercises can be performed either in the standing or lateral position. Fredericson et al. [26] described and compared the relative effectiveness of three common standing stretching exercises for the ITB (Figure 1). The difference between the stretches was mainly in the position of the upper body and trunk, while the lower part of the body remained unchanged. During all three stretches, the leg to be stretched is extended and adducted over the other leg. During the first stretch, stretch A (Figure 1A), the subject slowly flexes the trunk laterally to the opposite side of the stretched leg while exhaling. This movement is continued until a stretch is felt on the side of the hip around the greater trochanter. During the second stretch (stretch B, Figure 1B), additional movement of the upper limbs is added. Specifically, the hands are clasped overhead in the direction of lateral trunk flexion. The third standing stretch, stretch C (Figure 1C), is the same as stretch B, except that the subject extends the arms diagonally downward rather than overhead. In their intervention, Pepper et al. [27] also included a form of standing ITB–TFL complex stretch, which was similar to stretch B, described above. However, unlike Fredericson et al., they used a position in which the hip was externally rotated, in addition to extension and adduction. The additional external rotation of the hip may contribute to greater stretch, as it has been found that the stiffness of the ITB also increases with this motion [28]. 

Stretching the ITB–TFL complex in side-lying position has also been used in intervention studies [29,30]. Afshari et al. [29] studied the effects of the PNF (contraction–relaxation) stretching technique with the subject lying on his or her side, so that the side to be treated was facing upward and the knees were bent to 90 degrees. Meanwhile, the therapist stood behind the patient, placing one hand on the patient’s iliac crest and the other hand holding the knee. In the first phase of PNF stretching, the therapist moved the limb to the end of hip extension, adduction and external rotation, without producing any pain symptoms. After the contract–relax PNF technique, the patient was asked to press his foot on the therapist’s hand for five to eight seconds with a maximum voluntary contraction toward hip flexion, abduction, and internal rotation, while the knees remained flexed. In the third phase, after asking the patient to relax the muscles, the therapist brought the subject’s hip into the new range of extension, adduction, and external rotation. The PNF stretching cycle continued until the hip RoM did not increase. Kasunich [30], following a textbook recommendation by McConnel and Fulkerson [31], used a more passive form of ITB–TFL complex stretching performed by the patient without therapist involvement. The stretch began in the side-lying position with the affected leg elevated. In this position, the patient flexed the upper hip to 90 degrees to more easily grasp the upper foot with the hand. This was followed by active abduction and extension of the upper hip so that the heel of the foot rested against the upper buttock. Then the hip was allowed to drop into adduction and the opposite heel was placed on the upper knee for a greater stretch.

In addition to quite a few variations of ITB–TFL complex stretching, studies also vary in terms of the length of time a stretch is held and in the sets per stretching session. While Pepper et al. [27] described holding a stretch for only 15 s in each of the three bouts, Friede et al. [32] recommended that patients hold the stretch for 60 s and repeat it twice. In one study [24], participants also underwent an eight-week progressive stretching program that included four different stretching exercises for the ITB performed three times per week. For each exercise, the volume was progressed from 2 × 30 s stretching in the first four weeks, to 3 × 40 s stretching in the fifth and sixth weeks, and 4 × 40 s stretching in the last two weeks. However, a recent review [33] concluded that the increase in RoM was mainly related to the total time spent stretching per week, rather than the time spent stretching per session. A minimum of five minutes of stretching per week is required to achieve a significant improvement in RoM [33]. In addition, stretching performed at least five times per week has been shown to lead to further progress compared to a lower weekly frequency [33]. There is a lack of studies examining the long-term effects of ITB–TFL complex stretching. Friede et al. [32] asked their participants to perform a total of 28 min per week of stretching exercises at home for six weeks (two sets of 60 s duration, twice daily). On the other hand, McKay et al. [24] progressed their stretching exercise program weekly, in which the total duration of stretching exercises was 12 min per week during the first four weeks (four exercises, 30 s each, twice per day, three days per week), 24 min per week during the fourth to sixth weeks (four exercises, 40 s each, three times per day, three days per week) and 32 min per week during the sixth to eighth weeks (four exercises, 40 s each, four times per day, three days per week). When treating a runner with ITB tightness, Kasunich [30] in his case report also mentioned that ITB stretching was performed twice per day. Therefore, the currently available studies on the long-term effects of ITB–TFL complex stretching go far beyond the recommended general guidelines regarding the weekly frequency and duration of stretching per week.

**Figure 2:**
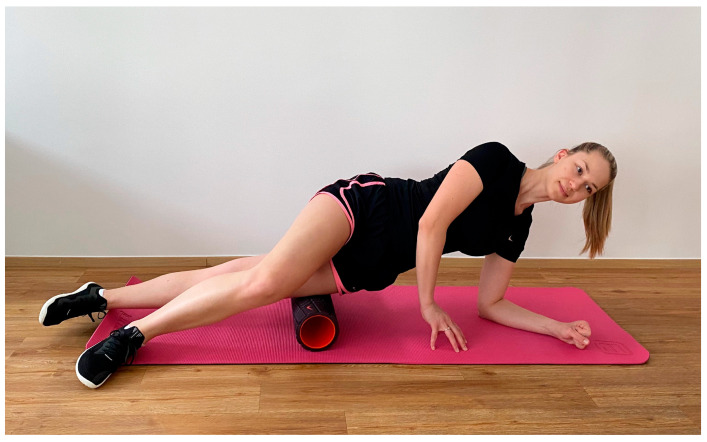
Typical positioning for ITB foam rolling.

To improve the flexibility of the ITB–TFL complex, increase RoM, and restore tissue extensibility, releasing techniques such as foam rolling have also been incorporated into the treatment of ITBS [18,34,35]. Foam rolling of the ITB–TFL complex typically involves positioning the subject’s body parallel to the floor and placing the foam roller between the floor and the thigh, with its long axis perpendicular to the long axis of the thigh (Figure 2) [27]. In order to apply adequate pressure to the area being treated, the subject must transfer their body weight to the roller and balance the weight with their hands and feet as needed [27]. For the treatment of ITB tissue, the roller should be moved from the greater trochanter to the lateral knee joint line [32,36,37]. Foam rolling study interventions for ITBS or ITB shortness management usually involve rolling over the ITB tissue only [29,32,38]. However, Park et al. [39] created a program of independent myofascial release using a foam roller for adult male cyclists with ITBS, treating the following tissues: Triceps surae, Tibialis anterior, Quadriceps femoris, Tensor fasciae latae, and Gluteus maximus. While in some studies [27] participants performed a more dynamic foam rolling, moving the body back and forth over the roller, in other studies [36,38] participants had to find the painful area or abnormally tender point and hold a static position for a period of time. Most studies that examined the effects of ITB–TFL complex release used the duration of a foam rolling session of one to five min [27,29,32,36,37], with the exception of Park et al. [39], whose foam rolling session lasted 20 min. A longer duration of their intervention is to be expected, since they treated five different muscles in one session. However, the duration of rolling over one muscle group in one session was four minutes, which is within the previously mentioned time frame of the other studies. In addition to foam rolling, other tissue-relaxing treatments mentioned in the studies include the Emmet technique [36], hands-on myofascial techniques addressing trigger points [32] and an osteopathic manipulative technique called counterstrain [40]. 

## 4. In Vitro and In Vivo Behavior Responses of Iliotibial Band to Stretching

The behavior of ITB during stretching has been the subject of several in vitro and in vivo studies. Fredericson et al. [26] studied the effects of three clinical stretches (i.e., final static positions of the stretches A, B, and C, as described above and shown in Figure 1) with kinematic analysis (using reflective markers attached to the skin), and reported a stretch of the ITB–TFL complex of 9.8–11.2%. However, others [41] pointed out that this study was unable to show where the stretch occurred within the ITB-TLF complex. Falvey et al. [41] showed that during hip adduction maximal voluntary contraction, the junction between the TFL and ITB was displaced by only 2 mm on average (resulting in a ITB lengthening < 0.5%), raising the question of the extent to which the ITB can be stretched in vivo.

A recent cadaver study showed that clinical-grade stretching of the entire complex resulted in greater elongation (4.45%) in the proximal region than in the distal (1.7%) and middle (1.42%) regions [42]. This is expected because the proximal section includes the TFL muscle, which is more compliant than the ITB [43]. Nevertheless, these findings indicate that middle and distal ITB regions are also subjected to elongation during clinical-grade stretching. Accordingly, an ultrasonographic study found a reduction in ITB width (suprapatellar level) from resting position (5.2 ± 0.8 mm) to the modified Ober’s test position (4.6 ± 0.6 mm), and a further reduction with an additional 3 kg load below the knee (3.9 ± 0.7 mm) [44], suggesting that clinical stretching may stress the ITB in addition to the TFL. While increased stiffness of the ITB has been observed in various stretched and active conditions (Ober test, TFL contraction, standing) compared to rest [28,45], a recent study found no acute effect of stretching (including foam rolling) on the stiffness of the ITB (mid-thigh and distal sections) in healthy participants measured in vivo at rest [27]. While it could be speculated that the stretching stimulus was inadequate (3 sets consisting of a 7 s submaximal contraction in hip abduction, followed by a 15 s stretch), this study suggests that typical clinical-grade stretching protocols may not affect ITB stiffness. 

In summary, typical clinical stretching exercises do cause some stretching of the ITB, while the predominant source of stretching is in the proximal portion, which includes the TFL muscle. Based on in vitro studies, ITB elongation with chronic stretching could be achievable, but clinicians must be aware that any observed improvements in hip RoM could be due to improved flexibility of the TFL. 

## 5. Iliotibial Band Syndrome Etiology and Risk Factors

This section discusses the rationale for including ITB stretching or “release” methods with regard to ITBS etiology and risk factors. Although the conclusions based on these studies are largely speculative, they may provide deeper understanding of the effects of stretching or release methods in ITBS patients. Traditionally, ITBS has been considered a friction syndrome resulting from rubbing of the ITB against the lateral femoral condyle during the knee flexion–extension cycle [23,46]. However, studies in cadavers have led to a new etiologic model. Because the ITB is anchored to the femur by fibrous strands [47], its movement in the sagittal plane is likely very limited. The ITB moves primarily in the medio-lateral direction, potentially pressing on underlying tissues. Accordingly, the currently prevailing theory suggests that compression is the primary cause of ITBS development and should be minimized [13]. The compressive force under ITB is positively related to the width of the lateral condyle. The lateral condyle can be thought of as the axis of a pulley, with one line of force pointing toward the ITB origin and one toward its attachment to the tibia [16]. The greater the angle between the two lines of force, the greater the compression component (see Friede et al. [16] for a detailed biomechanical description). Accordingly, patients with ITBS have been found to have slightly larger prominence of the lateral condyle compared with asymptomatic individuals [48]. However, because this factor is not modifiable and cannot be influenced by stretching, we will discuss below other factors that potentially influence compression forces, such as hip and knee alignment, ITB width and stiffness, and muscle activity.

One factor that may influence compressive forces is joint alignment. According to the model of Friede et al. [16], excessive hip adduction or knee varus should lead to an increase in compressive force. One study demonstrated increased dynamic knee varus in ITBS patients, as well as maximal varus velocity during running [49]. On the other hand, studies have shown both larger [50,51] and smaller [52,53] hip adduction angles during running in ITBS patients compared to controls. Stretching of the ITB–TFL complex could potentially increase hip adduction angle. Given the ambiguity regarding the influence of hip adduction on the development of ITBS, we cannot currently argue for either a positive or negative influence of stretching from this perspective. It is difficult to say how stretching the ITB–TFL complex would influence dynamic knee varus. A more compliant ITB–TFL complex could potentially even increase the knee varus if the hip angle is unchanged. If stretching also increased hip adduction, this could decrease dynamic varus. Given that knee valgus is also considered a risk factor in other knee overuse syndromes [54] and that the role of hip adduction in ITBS etiology is unclear, it is again difficult to say whether stretching would be beneficial or not. 

Another factor that has been explored with regard to the etiology of ITBS is the stiffness of the ITB. On the one hand, greater ITB stiffness could increase the compressive forces acting on the underlying tissue [16]. However, a recent study measuring ITB stiffness using shear wave elastography suggests that ITBS patients actually have lower ITB stiffness than asymptomatic control subjects [32]. Furthermore, ITB stiffness increased after intervention, while pain and lower extremity function improved significantly. Although stretching was part of the intervention, these results suggest that increased, rather than decreased, ITB stiffness may be associated with improved pain and function. 

In addition, running exercise has been shown to acutely reduce ITB stiffness (based on shear wave elastography) [45]. While it remains to be investigated whether excessive running training leads to a long-term reduction in ITB stiffness, this would be consistent with the above findings of lower stiffness in ITBS patients, and suggest that stretching may not be beneficial or even harmful for this population. A higher stiffness of connective tissues is thought to allow for greater elastic energy storage, and the ITB itself has been shown to be an important co-contributor to elastic energy return during running [55]. A recent study also showed an increase in energy cost associated with a decrease in Achilles tendon stiffness [56]. Although this has not yet been studied, it could be argued that a stiffer ITB could contribute to a more efficient running and consequently reduce the risk of injury. 

On the contrary, some studies have indicated that lower passive hip adduction range of motion is a risk factor for ITBS [57,58]. Although this could be seen as evidence that stretching might influence ITBS development, these aforementioned findings are based on case–control study designs. In addition, although Ober’s test (used in the above studies to determine hip adduction flexibility) results in stretching of the ITB [59], its outcome is unlikely to depend solely on the extensibility of the ITB–TFL complex. Rather, the results of the Ober test may be limited by tight gluteal muscles and hip joint capsule [60], which may have blurred the difference in hip adduction flexibility between ITBS patients and asymptomatic control subjects.

In summary, from the biomechanical point of view, there is little argument that ITB–TFL complex stretching is either beneficial or unfavorable in the treatment and prevention of ITBS. Namely, if the elongation of the ITB–TFL is assumed, some biomechanical risk factors may be resolved, while others could be exacerbated. Studies examining ITB stiffness as risk factor do not support stretching as a beneficial prevention/treatment method. It should be noted that this section is largely speculative, and provides only a theoretical summary of potential interplay between ITBS risk factors and treatment outcomes. The next section examines a more direct line of evidence available from intervention studies. 

## 6. Clinical Studies Examining the Effects of ITB Stretching and Other Methods Purported to Stretch or “Release” the ITB

Table 1 includes an overview of studies related to the primary research question. In subsequent paragraphs, the findings are discussed for each outcome measure separately.

### 6.1. Effects on Pain

There are a limited number of studies examining the effects of stretching or releasing the ITB–TFL complex on pain in people diagnosed with ITBS. McKay et al. [24] found a nonsignificant improvement in pain (as measured by the Numeric Rating Scale (NRS)) after an eight-week ITB stretching intervention in female runners with chronic ITBS (>three months). In addition, there was no statistical difference in pain improvement after the intervention between the stretching group, the conventional exercise group, and the experimental hip strengthening exercise group [24]. Similarly, Kasunich [30] documented an improvement in a 38-year-old female runner with low back and sacroiliac pain, which was attributed to ITB tightness, when extensive stretching was included in the treatment plan. However, they did not define the exact outcomes in which improvement occurred. Stretching the ITB–TFL complex may result in some improvement in pain; however, these improvements are neither statistically significant nor better than those observed with strengthening exercises. If pain improvement with stretching is similar to that with strength training, the latter may be a better treatment choice because it induces some additional adaptations not induced by stretching (such as an increase in muscle mass and strength, and a reduction in the risk of sports-related injuries) [61,62,63,64].

**Table 1:** Overview of included studies investigating effects of stretching and other release methods as treatment approach for ITBS.

Author (Year)	Population	Purpose	Intervention	Findings
Pepper et al., 2021 [27]	Healthy adults (18–50 years) with no ITBS history	To compare the immediate effects of stretching and foam rolling on ITB stiffness.	Stretching: Three bouts of a 7 s submaximal contraction in hip abduction followed by a 15 s stretch. FR: Five 3 min repetitions	No effects of either intervention on ITB stiffness, despite increases in hip RoM.
McKay et al., 2020 [24]	Female distance runners (19–45 years) with unilateral ITBS for at least 3 months	To assess the effectiveness of three different exercise regimens (stretching, conventional hip rehabilitation, and experimental exercises, which involve progressive increase in complexity) in female runners with ITBS.	Stretching (progressive stretching program included four ITB stretches that were held for 30–40 s and repeated 2–4 times, three times per week). Group B: Conventional exercise (focusing on hip muscles) Experimental hip strengthening exercise. The intervention lasted for eight weeks.	There were no statistical differences between the three groups. Nonsignificant improvement in pain was observed after stretching intervention. ITB stretching was reported to improve Y-balance test performance and movement quality in the single-leg mini-squat.
Afshari et al., 2023 [29]	Semi-elite athletes (20–40 years) with ITB shortness confirmed by the modified Ober’s test	To investigate the effect of active stretching techniques and self-myofascial release on improving the ITB flexibility and functional performance of athletes.	FR: foam rolling took about 3 min. PNF active stretching: contraction–relaxation technique for 3–5 min. Combination of FR/PNF	The mean of the active hip adduction RoM, single-leg hop test, lateral hop test, and vertical jump in all three groups increased significantly after the intervention compared to before. All three studied groups had similar changes over time, and no group was superior to the others.
Kasunich, 2003 [30]	Case report: long-distance runner (38 years) with low-back pain and sacroiliac pain and proposes ITB tightness as a possible causative factor	To investigate the effects of a multimodal therapy based on stretching and release methods on a single patient.	Chiropractic manipulative therapy, trigger point therapy and stretching of the ITB (once per day during first two weeks and twice per day during second two weeks, in side-lying position). The intervention lasted for 4 weeks.	The patient did not demonstrate much improvement until extensive stretching was included in the treatment plan. It is important to consider ITB tightness as a possible cause of low back and sacroiliac pain.
Friede et al., 2020 [32]	Recreational runners with ITBS and healthy controls (18–45 years)	To test ITB stiffness and isometric hip muscle strength in a sample of subjects clinically diagnosed with ITBS for comparison with a healthy control group, and to assess the effectiveness of a multimodal training program in strengthening the hip abductor and external rotator muscles and modulating ITB tone.	The intervention lasted for 6 weeks and consisted of: myofascial techniques addressing trigger points, strengthening exercises (for gluteal muscles and hip external rotators), stretching (twice a day, two sets of 60 s duration, 30 s inter-set break), foam rolling (three times for 60 s, 30 s break between sets). Intervention also consisted of measures aiming to improve neuromuscular control and lower extremity alignment during gait and running.	ITB tension is not increased in the affected legs of runners with ITBS compared to the healthy leg or a physical active control group, respectively. Following six weeks of physiotherapy, hip muscle strength (all directions but abduction), pain and lower extremity function were significantly improved. ITB stiffness was found to be increased compared to baseline measurements.
Sharp et al., 2012 [36]	Asymptomatic male non-professional rugby players (19–30 years)	To compare the relative effectiveness of two myofascial release techniques for the ITB: self-myofascial release (foam rolling), and Emmett technique.	Emmet technique used on the “ITB site” and the “sartorius and ITB site” (lasted for 5 min). FR: focused on TFL and ITB area, left and right side were treated (there was a maximum time of 60–90 s allocated for each region). Control: no intervention.	No significant improvements in active hip adduction RoM were observed after foam rolling. Emmet technique appeared to be more effective in hip RoM increase (2% increase in hip RoM after FR, 70% increase after Emmet technique). No significant effect of FR and Emmett technique on vertical jump height during countermovement jump was observed.
Park et al., 2022 [39]	Men cycling club members (20–45 years) with ITBS	To investigate the effect of one-time self-myofascial release using a foam roller via special tests, visual analog scale (VAS), and exercise performance on adult male cycling club members diagnosed with ITBS.	After the first 10 km cycling course, the control group had a static rest for 120 min. The FR group conducted the intervention using a foam roller for 20 min after a static rest of 100 min, and then both groups underwent a post-cycling special test after the second cycling on the same course.	Significant differences were observed in the FR group in VAS through Nobel’s compression test, ITB flexibility through Ober’s test, and VAS and power while cycling. No significant difference was observed in HR, cadence, and record time.
Pedowitz, 2005 [40]	Case report: 30-year-old distance runner with ITBS	To investigate the potential of osteopathic manipulative technique (OMT) called counterstrain on a single-case basis.	The intervention lasted for 2 weeks with OMT applied every 2 to 3 days.	One week after intervention the patient reported that he had been feeling well and had returned to his normal, full running activity by day. He also stated that he had been free of pain and feeling happier overall. His ability to perform the regular activities of daily life had improved.
Else and Moodley, 2010 [38]	Active runners or cyclists with ITBS and active or latent trigger points in the ITB (18–60 years)	To determine whether foam roller treatment of the ITB can be used as an effective treatment for ITBS in cyclists and runners and whether it is better to be used alone or in conjunction with spinal manipulation.	Intervention lasted for 3 weeks (6 treatment consultations). Group one: combination of both treatments. Group two: foam rolling (they held their weight over the foam roller on painful area for minimum of 120 s). Group three: lumbar spinal manipulation to the restricted segments.	Combination group showed the best improvement consistently across all forms of measurement. Group three had the smallest increase in overall improvement subjectively and objectively. All three treatment protocols were equally effective in treating ITBS as demonstrated by the statistically significant results.
Vaughan et al., 2014 [37]	Student asymptomatic population (both genders, mean age of 26.1 ± 6.7 years)	To investigate the effects of the application of a foam roller for three minutes to the right iliotibial band (ITB) of asymptomatic participants.	A three-minute session on the foam roller.	Results demonstrate a statistically significant increase in the pain pressure threshold at the lower thighimmediately post-bout; however, the difference was ameliorated five minutes later.
Mayer et al., 2020 [65]	Experienced (regarding FR) and nonexperienced athletes (healthy; both genders; 18–65 years)	To investigate muscle-specific and connective tissue-specific responses after FR in recreational athletes with different foam rolling experience.	FR: 5 trials per 45 s of foam rolling with 20 s of rest between each trial.	In experienced athletes, tissue stiffness of the ITB revealed a significant decrease of 13.2% at post-intervention (t1 = after 0 min) and 12.1% 6 h after intervention (=t3). In nonexperienced athletes, a 6.2% increase in stiffness was found at t1, which was not significantly different to baseline. For both groups, no significant ITB stiffness changes were found at further time points (30 min, 6 h or 24 h after intervention).

Regarding foam rolling, the research is fairly consistent, and indicates acute and long-term improvements in pain scores in ITBS patients. Park et al. [39] examined the acute effects of a 20 min foam rolling treatment in adult male cyclists with ITBS, and found a significant reduction in pain felt during the second round of cycling after foam rolling compared to pain felt during the first round of cycling performed without prior to the intervention. In addition, they noted a significant decrease in Visual Analogue Scale (VAS) scores measured by the Nobel compression test after the second cycling round. Immediate effects of foam rolling on pain were examined by Vaughan et al. [37], who reported a significant increase in pain pressure threshold (PPT), measured at the lower part of the ITB, after 3 min of foam rolling intervention. However, this increase was not maintained 5 min later. In addition, the participants were asymptomatic and did not have ITBS. The results might have been different if the intervention had lasted longer and additional muscle groups had been trained, as done by Park et al., or if the subjects were diagnosed with ITBS. In contrast to Vaughan et al., Else and Moodley [38], who investigated the effect of foam rolling on PPT in ITBS patients, found a significant improvement in pain threshold at the seventh visit (after six treatment sessions). Although they did not mention the exact time frame between the last treatment session and the measurements taken at the seventh visit, we can assume that the improvement in PPT was maintained several days after the last treatment. Furthermore, Else and Moodley found a significant improvement in subjective pain perception (measured with NRS) after six treatment sessions of foam rolling. Significant improvements in subjective perceived pain (measured with VAS) were also observed in runners with ITBS after six weeks of multimodal treatment consisting of stretching exercises, foam rolling and other interventions to reduce ITB tightness, strengthening exercises for the hip stabilizing muscles, neuromuscular control exercises, and exercises to improve lower extremity alignment [32]. However, because of the multimodal treatment, we cannot attribute these results to foam rolling or stretching alone. The available studies that have examined the effects of Foam Rolling on pain in ITBS patients have fairly consistent results, indicating significant improvement in pain after treatment. However, it is not yet known whether these positive effects are sustained over the long term and contribute to the resolution of ITBS. If pain relief does not persist after foam rolling, this intervention is probably not the best solution for treating ITBS as long as the goal is pain relief. Based on current studies, athletes with ITBS who cannot avoid future competitions would likely benefit most in terms of acute pain relief from foam rolling, as it would allow them to compete with less pain and thus achieve better results.

### 6.2. Effects on Stiffness

As far as we know, there are no studies reporting a reduction in ITB stiffness after stretching or other interventions that purportedly “relieve” the ITB in ITBS patients. On the contrary, distal ITB stiffness was found to increase by 13.5% in ITBS patients after a six-week multimodal program that included stretching and foam rolling, in addition to other treatment modalities (hip muscle strengthening, myofascial techniques to treat trigger points, and neuromuscular control training) [32]. However, the observed increase in ITB stiffness could be a result of the strengthening exercises for the hip stabilizing muscles that were also part of the multimodal treatment. Hip strengthening exercises could increase the resting tone of the muscles inserting into the ITB, which in turn could cause an increase in ITB stiffness [32].

We have found conflicting results regarding the outcomes of interventions aimed at reducing ITB stiffness performed in healthy individuals. In a study by Pepper et al. [27], neither stretching nor foam rolling altered ITB or TFL stiffness. Because only a single bout of stretching and foam rolling was performed in their study, the insufficient duration of the intervention could explain these results. Nevertheless, the results suggest that stretching and foam rolling have no effect on the stiffness of the ITB and TFL in healthy subjects [27]. In contrast, “releasing” methods have been suggested to decrease ITB stiffness in other studies [65]. However, the decrease in ITB stiffness observed after a single foam rolling intervention in a study by Mayer et al. [65] after a single episode of foam rolling intervention was significant only in recreational athletes experienced with foam rolling. In addition, a significant decrease in ITB stiffness was only observed immediately and 6 h after the intervention, whereas no significant changes were observed 24 h after foam rolling.

There is some evidence that foam rolling results in short-term reductions in ITB stiffness in healthy experienced athletes [65]. However, an increase in ITB stiffness has been observed in ITBS patients after multimodal treatment [32]. While one might expect a worsening of symptoms due to increased ITB stiffness and resulting increased compressive forces, the opposite was observed. Despite the significant increase in ITB stiffness, symptoms improved significantly after six weeks of multimodal treatment [32]. Therefore, the question arises as to whether a rehabilitation program for ITBS should instead aim to increase ITB stiffness. In addition to alleviating symptoms, increasing ITB stiffness could also improve energy storage during running [55], and therefore be a reasonable long-term goal in ITBS rehabilitation. 

### 6.3. Effects on Hip RoM

In male cyclists diagnosed with ITBS, one bout of foam rolling has been shown to significantly increase hip adduction RoM (assessed in the Ober’s test position), which may be due, at least in part, to reduced stiffness of the ITB [39]. To our knowledge, there are no other studies that have examined changes in hip mobility after foam rolling or stretching interventions in ITBS patients. However, there are studies in healthy subjects that have somewhat contradictory results. Pepper et al. examined the immediate effects of foam rolling and ITB–TFL complex stretching. They found an increase in passive hip adduction RoM after the intervention; however, this change was small (0.8°) and also occurred in the control group, which could be due to measurement error and is probably not clinically relevant [27]. On the other hand, a study by Afshari et al. [29] measured active hip adduction RoM in semi-elite athletes with ITB shortness (confirmed with modified Ober’s test), with similar improvements observed over time immediately following ITB foam rolling, active PNF stretching, and the combination of both treatments. In a study conducted on healthy rugby players by Sharp [36], no significant improvements in active hip adduction (RoM) were observed after foam rolling. Furthermore, only a 2% increase in hip RoM was observed after foam rolling intervention, while the Emmett technique appeared to be markedly more effective, with a 70% increase after the intervention [36].

Based on the only study found that assessed the effects of ITB-releasing techniques on hip mobility in ITBS patients [39], we might recommend the inclusion of foam rolling in an ITBS rehabilitation program if our goal is to improve hip RoM. However, these results may not extend beyond the study population (cyclists). Given the inconsistent results of studies conducted in a healthy population, we can speculate that similar discrepancies would occur if more studies were conducted in ITBS patients. In addition, Park et al. [39] measured improvements in ITB flexibility through changes in hip adduction RoM in Ober’s test. Considering that hip adduction in the Ober’s test position may be limited not only by the ITB, but also by gluteal muscles and hip joint capsule tightness [59,60], the results measured through the Ober’s test do not necessarily indicate an increase in hip adduction solely due to ITB release. Furthermore, the existing ambiguity about the influence of hip adduction on the development of ITBS (see Section 4) raises the question of whether it is appropriate to include methods to increase hip ROM in the rehabilitation program of ITSB patients.

### 6.4. Effects on Function and Performance 

Research shows the positive effects of ITB complex stretching and releasing methods on improving function and performance in ITBS patients. Park et al. [39] found that a one-time foam rolling program in adult male cyclists with ITBS resulted in an increase in pedaling power during cycling. The intervention also tended to increase 10 km cycling performance, but this did not reach statistical significance [39]. In addition, 8 weeks of ITB stretching was reported to improve Y-balance test performance and movement quality in the single-leg mini-squat; however, the study report does not indicate whether stretching was more or less effective than the hip strength program [24]. Foam rolling and stretching exercises combined with hip-strengthening exercises in a six-week multimodal program for recreational runners with ITBS resulted in significant improvements in Lower Extremity Functional Scale (LEFS) scores after the intervention [32]. The osteopathic manipulative technique called counterstrain is another release method that showed improvement in function in a 30-year-old man diagnosed with ITBS after two weeks of treatment [40]. One week after treatment, the patient reported that he had returned to normal, full running activity, and that his ability to perform regular activities of daily living had improved. In addition, the patient was still able to walk and exercise without limitations ten weeks after starting treatment [40]. On the other hand, studies conducted on an asymptomatic population have inconsistent findings. While Sharp [36] observed no significant effect of foam rolling and the Emmett technique on vertical jump height during countermovement jumping, Afshari et al. [29] found a significant increase in the mean of single-leg hop test, lateral hop test and vertical jump after foam rolling, PNF active stretching technique and the combination of both interventions. Furthermore, they emphasized that all of the interventions studied showed similar changes over time, and no one group was superior to the others.

While one-time interventions suggest that the observed acute performance improvements are indeed due to foam rolling alone, these results can only be generalized to the population of male cyclists with ITBS, as only one study [39] examined acute effects on performance in ITBS patients. Another issue arises with some long-term interventions, where it is difficult to attribute functional improvements solely to the interventions, as some studies [24,40] asked patients to avoid painful activities or make changes in running shoes and training surface, which could also influence the observed outcomes. In studies in which the intervention consisted of strengthening exercises in addition to stretching and foam rolling [32], the observed improvements are also unlikely to be the result of releasing methods alone. Therefore, the effects of stretching and releasing methods on function and performance are questionable, especially in terms of long-term improvements.

## 7. Conclusions

Stretching the ITB–TFL complex has been a part of ITBS rehabilitation for years. This review critically evaluated the currently available evidence for or against the inclusion of stretching or other “release” methods for ITBS patients. In vivo and in vitro studies show that typical clinical stretching exercises do provide some stretching of the ITB, while the predominant source of the stretch is in the proximal portion, which includes the TFL muscle. Examination of ITBS etiology and risk factors offers little evidence on how stretching the ITB–TFL complex would affect ITBS risk or treatment. On the one hand, a stiffer ITB could increase soft tissue compression in the painful region; on the other hand, a stiffer ITB together with a stronger TFL could improve frontal knee kinematics. There is some evidence that stretching and “release” methods may provide some benefit in terms of pain reduction, increase hip ROM and provide functional improvements. However, the studies evaluating stretching or “releasing” in isolation have almost exclusively assessed acute effects only. Evidence from long-term intervention studies suggests the use of stretching as part of a multimodal treatment; however, the extent to which stretching within a multimodal program contributes to ITBS treatment is currently unclear. Finally, recent evidence suggests that ITBS patients do not have stiffer ITB, and that an increase (rather than a decrease) in ITB stiffness may be associated with favorable treatment outcomes. In summary, the current literature provides some evidence of the favorable acute effects of stretching or “releasing” on pain and function, supporting the use of these methods in early rehabilitation. According to other recent comprehensive reviews, stretching should be incorporated at the beginning of the treatment program (weeks ~0–4) in cases of RoM restrictions, while hip strengthening is the main goal in the long-term. We cannot completely refute stretching and releasing of the ITB as a valuable component of long-term rehabilitation of ITBS; however, future studies should compare intervention programs with and without stretching to clarify whether stretching adds benefits to other components (e.g., activity modification, strengthening). In addition, it has to be emphasized that we detected no reports on any negative effects of stretching or “releasing” the ITB. Although there is some indirect evidence (based on ITBS risk factors and changes in ITBS stiffness) suggesting that stretching and releasing the ITB may interfere with ITBS rehabilitation, there is no direct empirical evidence to support this.

## Data Availability

No new data were generated for this article.
